# Relationship of Topology, Multiscale Phase Synchronization, and State Transitions in Human Brain Networks

**DOI:** 10.3389/fncom.2017.00055

**Published:** 2017-06-30

**Authors:** Minkyung Kim, Seunghwan Kim, George A. Mashour, UnCheol Lee

**Affiliations:** ^1^Department of Physics, Pohang University of Science and TechnologyPohang, South Korea; ^2^Center for Consciousness Science, University of Michigan Medical SchoolAnn Arbor, MI, United States; ^3^Department of Anesthesiology, University of Michigan Medical SchoolAnn Arbor, MI, United States

**Keywords:** emergence, explosive synchronization, state transition, anesthesia, brain network, consciousness, Kuramoto model

## Abstract

How the brain reconstitutes consciousness and cognition after a major perturbation like general anesthesia is an important question with significant neuroscientific and clinical implications. Recent empirical studies in animals and humans suggest that the recovery of consciousness after anesthesia is not random but ordered. Emergence patterns have been classified as progressive and abrupt transitions from anesthesia to consciousness, with associated differences in duration and electroencephalogram (EEG) properties. We hypothesized that the progressive and abrupt emergence patterns from the unconscious state are associated with, respectively, continuous and discontinuous synchronization transitions in functional brain networks. The discontinuous transition is explainable with the concept of explosive synchronization, which has been studied almost exclusively in network science. We used the Kuramato model, a simple oscillatory network model, to simulate progressive and abrupt transitions in anatomical human brain networks acquired from diffusion tensor imaging (DTI) of 82 brain regions. To facilitate explosive synchronization, distinct frequencies for hub nodes with a large frequency disassortativity (i.e., higher frequency nodes linking with lower frequency nodes, or vice versa) were applied to the brain network. In this simulation study, we demonstrated that both progressive and abrupt transitions follow distinct synchronization processes at the individual node, cluster, and global network levels. The characteristic synchronization patterns of brain regions that are “progressive and earlier” or “abrupt but delayed” account for previously reported behavioral responses of gradual and abrupt emergence from the unconscious state. The characteristic network synchronization processes observed at different scales provide new insights into how regional brain functions are reconstituted during progressive and abrupt emergence from the unconscious state. This theoretical approach also offers a principled explanation of how the brain reconstitutes consciousness and cognitive functions after physiologic (sleep), pharmacologic (anesthesia), and pathologic (coma) perturbations.

## Introduction

How does the brain reconstitute the capacity for consciousness and cognition after a major perturbation like general anesthesia? What determines reversibility in some states (e.g., sleep) and irreversibility in others (e.g., coma)? The underlying mechanism of the reconstitution of brain function is poorly understood despite significant neuroscientific and clinical implications. Anesthesia has been used as a tool to inhibit spontaneous brain activities and reversibly suppress consciousness but more recently has been used to investigate the recovery process from unconsciousness. Recent empirical studies demonstrated that brain recovery from anesthetic-induced unconsciousness is not random, but ordered. Hudson et al. found that during the emergence from anesthesia, brain dynamics pass through an ordered sequence of states that is different from a random walk (Hudson et al., 2014). Furthermore, diverse emergence patterns have been observed from the electroencephalogram (EEG) of humans. For example, Hight et al. reported two distinct emergence patterns in general anesthesia (Hight et al., 2014). One showed progressive spectral changes of EEG before the response, while the other showed no explicit change of EEG spectral properties before the abrupt return of responsiveness. Chander et al. classified the emergence patterns into four types based on the spectral behaviors of EEG such as delta (1–4 Hz) and alpha/spindle (8–14 Hz), as well as different levels of pain (Chander et al., 2014). These emergence patterns can be qualitatively described as “progressive and earlier state transition” and “abrupt but delayed state transition.” However, previous studies examined local field potentials and frontal EEG rather than global brain activities. Lee et al. identified network recovery properties in healthy individuals emerging from anesthesia that followed similar patterns, suggesting the possibility that differential network principles account for various behavioral phenotypes (Lee et al., 2011).

The synchronization process has been studied with cat and human brain networks investigating the role of hub, modular structure, and global network structure (Honey and Sporns, 2008; Breakspear et al., 2010; Gómez-Gardeñes et al., 2010; Cabral et al., 2011; Villegas et al., 2014; Hellyer et al., 2015; Schmidt et al., 2015; Váša et al., 2015; Finger et al., 2016). However, these studies were limited to progressive synchronization and did not address delayed or abrupt synchronization, which is potentially applicable to delayed anesthetic emergence. In this study we compare, for the first time, the distinct synchronization processes in a human brain network under progressive and abrupt synchronization conditions. Our main objective is to understand distinct emergence patterns in terms of network synchronization rather than model state-specific EEG signatures, *per-se*. Because temporal coordination is a necessary condition for information integration and transmission across brain regions, the recovery pattern of the brain network synchronization may reflect the recovery pattern of consciousness.

In this simulation study, we modeled the potential network mechanisms for these archetypal emergence patterns (“progressive and earlier” and “abrupt but delayed”) by assessing synchronization patterns in computational models based on neuroanatomically-derived human brain networks. We implemented the Kuramoto model, a simple oscillatory model, in a human brain network with 82 nodes (including cortical and subcortical areas) to simulate the dynamic interactions among brain regions. To facilitate the delayed but abrupt transition, we applied the principle of explosive synchronization, derived from network science, as a potential mechanism for the discontinuous transition from a desynchronized to synchronized state (Gómez-Gardeñes et al., 2011). High frequency disassortativity (Leyva et al., 2013b; Zhu et al., 2013; Skardal and Arenas, 2014) was applied to the human brain network in order to suppress giant synchronization cluster formation (Zhang et al., 2014, 2015). This network configuration primarily prohibits hubs from synchronizing, which leads to a delay in synchronization that reaches a critical point of abrupt global synchronization.

We furthermore compared distinct synchronization processes between progressive and abrupt transitions on the scale of the individual node, clusters, and global network structure. We demonstrate that the distinctive synchronization processes are significantly determined by the underlying brain network structure with a given frequency configuration. This approach could provide a principled explanation of how brain networks reconstitute regional activities during progressive and abrupt emergence at a network level, which could be applied to recovery from physiologic (sleep), pharmacologic (anesthesia), and pathologic (coma) states of unconsciousness.

## Methods

### Network model

We used a simple phase oscillator model, the Kuramoto model (Kuramoto, 1984), in a group-averaged anatomical brain network to simulate the dynamic behavior of two different types of emergence patterns.

The Kuramoto model is defined as the following:

(1) $$\theta_{i}=\omega_{i}+\lambda \sum_{j=1}^{N}A_{ij}\text{ sin}(\theta_{j}-\theta_{i}),i=1,2,\ldots N$$

Here, θ_*i*_ is the phase, ω_*i*_ is the initial angular frequency of i_*th*_ oscillator, and λ is the coupling strength between all connected nodes. *N* is the total number of nodes and *A*_*ij*_ is the adjacency matrix, which is an anatomical brain network structure. The anatomical brain network was acquired from group-averaged diffusion tensor imaging (DTI) with 82 nodes, including cortical and subcortical areas (Van Den Heuvel and Sporns, 2011).

### Network configuration

Initial phases randomly distributed between (−π, π) and specific frequency distributions for progressive and abrupt transition types were assigned to the nodes. In this simulation, we assume that different initial frequency distributions reflect different regional brain dynamics. According to explosive synchronization, the initial frequency distribution within the network topology may determine the synchronization path from the desynchronized state. We used a Gaussian distribution with the mean 10 Hz and variance 0.2 Hz to simulate the alpha bandwidth of human EEG activity (Moon et al., 2015). Here, we considered only the alpha frequency band (9 to 11 Hz), because the alpha frequency band shows significant and consistent global connectivity changes along with state changes induced by diverse anesthetics (Lee H. et al., 2013; Blain-Moraes et al., 2014; Kim et al., 2016). We generated 100 frequency configurations of Gaussian distribution to observe canonical behaviors of progressive transition. To simulate the abrupt transition, we first selected 15 nodes (18% of all nodes) with high degrees (i.e., high number of connections) as hub nodes on the basis of rich club organization using degree k = 21 (Van Den Heuvel and Sporns, 2011). These hub nodes include thalamus, hippocampus, putamen, superior frontal, superior parietal, precuneus, and insula of both hemispheres, which are potentially related to consciousness (Bogen, 1995; Martuzzi et al., 2010; Ku et al., 2011; Spoormaker et al., 2012) and important for inter-modular synchronization in human brain networks (Schmidt et al., 2015). We used different frequency distributions for the hub nodes (Gaussian distribution with mean 10.3 Hz and variance 0.05 Hz for 6 nodes; Gaussian distribution with mean 9.7 Hz and variance 0.05 Hz for 9 nodes), which can be one way to suppress the formation of a giant synchronization cluster in the system by inducing large frequency mismatches between high degree nodes (Zhu et al., 2013). In particular, we assigned the relationship between node degree and frequency a V-shape, which is similar to a previous study of explosive synchronization (Leyva et al., 2013b). We also calculated the frequency disassortativity (ρ_f_), defined as a Pearson correlation between node frequency and the average frequency of neighbor nodes, in order to achieve a more robust occurrence of abrupt transitions (Li et al., 2013). Large frequency disassortativity enhances the frequency mismatches between neighbor nodes and makes it possible to overcome the homogeneity of the network structure itself (Boccaletti et al., 2016). We generated 100 frequency configurations with large values of frequency disassortativity (ρ_f_ < −0.3) to analyze the characteristics of abrupt transition. Gaussian distribution with mean 10 Hz and variance 0.2 Hz with large frequency disassortativity (ρ_f_ < −0.3) and various frequency configurations with diverse frequency disassortativity values were also simulated for a comparison of the robustness (Figure S1). See Table 1 for an explanation of network terminology.

**Table 1:** Glossary of terms.

**Keywords**	**Descriptions**
Node Degree	The number of edges/links connected to a node in a network.
Hub	In this study, a node that has a high degree is defined as a hub node. Hub structure plays a crucial role in communication and information transmission in the brain.
First-order phase transition	Discrete changes from incoherent to synchronized state or vice versa, as the coupling strength of coupled oscillators increases or decreases, respectively. A more continuous change is referred to as a “second-order phase transition.”
Explosive (or abrupt) synchronization	A phenomenon characterized by first-order phase transition between incoherent and synchronized states in a network of coupled oscillators. The key mechanism of explosive synchronization is to suppress the formation of a giant synchronization cluster in a network, mainly inhibiting the hub synchronizations.
Progressive synchronization	A phenomenon characterized by a second-order phase transition between incoherent and synchronized states in a network of coupled oscillators. The hub node dominates the synchronization process by entraining the neighbor nodes.
Frequency disassortativity	A tendency for nodes oscillating at higher frequencies to connect with nodes at lower frequencies, or vice versa. Large frequency disassortativity contributes to generating the network conditions for explosive synchronization.

### Synchronization measures and computation

We numerically solved the differential equations of the Kuramoto model using the 4th order Runge-Kutta method with 1,000 discretization steps. The first half of the time series was discarded and the last 15 of 30 s were used for each simulation. The sampling rate was 1,000 Hz and the coupling strength λ increases from 0 to 0.4 with δλ = 0.002. In order to observe the dynamics of the functional network of each λ, we calculated the average pairwise synchrony between node *i* and *j*, *D*_*ij*_, defined as

(2) $$D_{ij}=A_{ij}\frac{1}{\Delta t}\left|\sum_{\tau }^{\tau +\Delta t}e^{i\left[\theta_{i}\left(t\right)-\theta_{j}(t)\right]}\right|$$

which is a symmetric phase synchronization matrix. Using the *D*_*ij*_, we can obtain an order parameter to estimate the level of global synchronization, r_link_,

(3) $${\text{r}}_{\text{link}}=\frac{1}{2N_{l}}\sum_{i,j}D_{ij}$$

where *N*_*l*_ is the total number of links. We also examined the synchronization level of each node, with the local order parameter represented as,

(4) $${\text{r}}_{\text{i}}=\frac{1}{2n_{i}}\sum_{j\;\in \;nn_{i}}D_{ij}$$

where *n*_*i*_ is the number of links connected with node i. We used median values for global and local order parameters of 100 configurations for the analysis in order to avoid the confound of outliers. With r_i_, we compared synchronization processes of two transitions at the individual node level.

### Synchronization cluster analysis

After exploring the behaviors of synchronization processes at the individual node level, we next investigated the synchronization process at the cluster level. One of the significant differences between progressive and abrupt transitions is the process of cluster merging. Therefore, we examined the process of synchronization cluster formation in the two transition types. We constructed the binary synchronization matrix *S*_*ij*_ defined as

(5) $$\{\begin{array}{ccc} S_{ij} & = & 1,\;if\;D_{ij}>0.95\\ S_{ij} & = & \text{​​​​​​​}0,\;otherwise \end{array}$$

We considered two nodes i and j as synchronized if *D*_*ij*_ is larger than 0.95 (Zhang et al., 2014). The synchronization threshold of 0.95 between connected links in the human brain network demonstrated distinctive changes in terms of the number of clusters and the size of a giant cluster for both transition patterns, and revealed the critical states for each transition pattern. With the matrix *S*_*ij*_ for each coupling strength λ, we calculated the number of synchronization clusters, *N*_*C*_, and the size of a giant synchronization cluster, GC, which is the largest synchronization cluster among all clusters. We then took the median of 100 configurations for each transition.

### Relationship between structure and dynamics

We examined the global relationship between structure and dynamics during both transitions to grasp the detailed synchronization process within the network topology. We first calculated the Spearman correlation between degree and median local order parameter over 100 configurations of each λ. We then divided the brain network into 5 subgroups with degree order; (1) 4 ≤ k < 11, (2) 11 ≤ k < 14, (3) 14 ≤ k < 18, (4) 18 ≤ k < 22, (5) 22 ≤ k < 32 to understand the dynamics of hub and peripheral nodes within the network structure. Each subgroup has at least 15 nodes. In one configuration, the synchronization level of each subgroup (r_s_) was acquired by taking the average of r_i_ within a subgroup. We then took the median of the average values over the repeated 100 configurations. We compared the synchronization level and rank among subgroups to reveal further details of the synchronization process for every λ associated with topology.

### Reconstitution order of brain region

We calculated the difference between first coupling strength values λ_r_i_>0.2_ and λ_r_i_>0.8_ satisfying r_i_ ≥ 0.2 and r_i_ ≥ 0.8 to investigate the reconstitution order of brain regions. The difference between the coupling strengths of each node was deemed to be the integration duration τ from a low synchronization level in an unconscious state to a high synchronization level in a conscious state. The given thresholds are empirically observed synchronization levels (Kim et al., 2016).

(6) $$\tau_{\text{i}}=\lambda_{r_{i}>0.8}-\lambda_{r_{i}>0.2}$$

We then ranked the 82 regions in terms of τ_i_ and repeated it over 100 network configurations. Within each transition, we compared the reconstitution orders among 82 regions and among 10 sub-regions (Table S1). We performed the Kruskal-Wallis test with multiple comparisons considering *p* < 0.05 as a significant difference among the regions with Bonferroni corrections. For the comparison of reconstitution orders of each region between two transitions, we used Wilcoxon rank sum test and defined the nodes with *p* < 0.05 as the brain regions with significantly different reconstitution orders between progressive and abrupt transitions. From this analysis, we could predict the regional recovery process in a transition as well as the differences of the global network recovery process between progressive and abrupt emergence patterns.

## Results

### Network configurations define progressive and abrupt transitions

**Figure 1:**
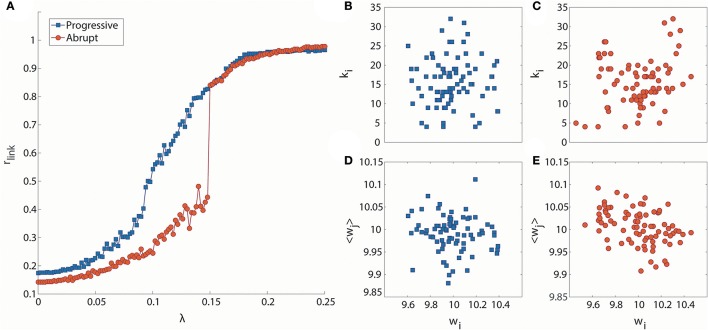
Distinct phase synchronization patterns in computational models of the human brain. **(A)** Exemplary cases for progressive (blue) and abrupt (red) transitions. The abrupt transition shows a sharp increase of the order parameter at λ = 0.148. The relationships between initial frequencies and node degrees for **(B)** progressive and **(C)** abrupt transitions are presented. The abrupt transition has a V-shape in the relationship. The relationships between initial frequencies and average frequencies of the nearest neighbor nodes are also presented for **(D)** progressive transition (ρ_f_ = −0.098) and **(E)** abrupt transition (ρ_f_ = −0.430).

Figure 1A presents two exemplary cases of progressive and abrupt transitions in a brain network as coupling strength increases. The distinctive patterns, progressive (blue), and abrupt (red), of global order parameters, r_link_, for both transitions are clear. The r_link_ of the progressive transition continuously increases from an unsynchronized to synchronized state, but the r_link_ of the abrupt transition jumps discontinuously at λ = 0.148, which is suggestive of a process involving explosive synchronization. Figures 1B–E shows the different initial network configurations for the progressive and abrupt transitions. The relationships between initial frequencies and degrees for 82 nodes are shown in Figures 1B,C. For the abrupt transition, we assigned a V-shape to the relationship between frequency and node degree, in accordance with a previous study (Leyva et al., 2013b). The V-shape relationship yields large frequency mismatches between high degree nodes, which inhibit the formation of giant synchronization clusters. The relationships between frequencies and the average neighbor frequencies are illustrated in Figures 1D,E. The frequency disassortativities are −0.098 and −0.430 for progressive and abrupt transitions. The large frequency disassortativity (ρ_f_ < −0.3) generates a tendency for a higher frequency node to have lower frequency neighbor nodes. These frequency mismatches applied to the nodes are more likely to produce abrupt synchronization in a network (Li et al., 2013).

### Global and local synchronization processes for progressive and abrupt transitions

**Figure 2:**
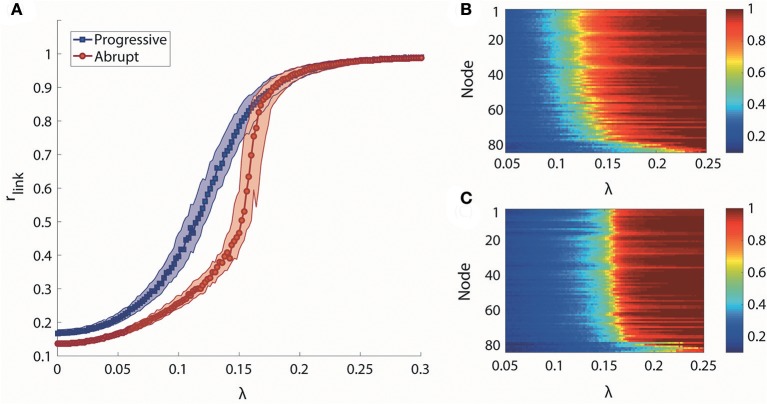
Global and local synchronization for progressive and abrupt transitions. **(A)** Median global order parameter of progressive (blue) and abrupt (red) transitions for 100 frequency configurations. Colored area indicates the 25–75% values of r_link_ of 100 configurations. Local order parameter r_i_ of **(B)** progressive and **(C)** abrupt transition. The nodes are aligned with descending order of degree from top to bottom. Color indicates the value of r_i_.

We took the median of r_link_ for 100 frequency configurations to observe the canonical behaviors for each transition type. The median r_link_ as a function of λ is shown in Figure 2A. Under the progressive transition condition (blue), the r_link_ increases gradually in all steps of λ. In the abrupt transition (red), the global synchronization is relatively delayed for a long period before a major change, followed by the steep increase of median r_link_ within a short range of coupling strength (around λ = 0.16). This delay was expected from the initial network configurations of the abrupt transition, which prohibits the network from being globally synchronized. Figures 2B,C shows how the higher and lower degree nodes were differentially synchronized during progressive and abrupt transitions. If we define a node with the local order parameter r_i_ = 0.8 as synchronized, the data demonstrate that, in the progressive transition, the sequence of the synchronization process correlates with the node degree. In other words, highly-connected hub nodes are synchronized earlier than less-connected peripheral nodes. By contrast, in the abrupt transition, the local synchronization of nodes across the network takes place suddenly when a critical threshold of coupling strength is crossed. This implies that the network topology itself, represented as the node degree, has influence on the synchronization level in the progressive transition, whereas the effect of network topology is suppressed by the frequency configurations during the abrupt transition before a critical level. Thus, distinctive synchronization processes for two types of transition are observed at the individual node level of the brain network.

### Synchronization cluster formations for progressive and abrupt transitions

**Figure 3:**
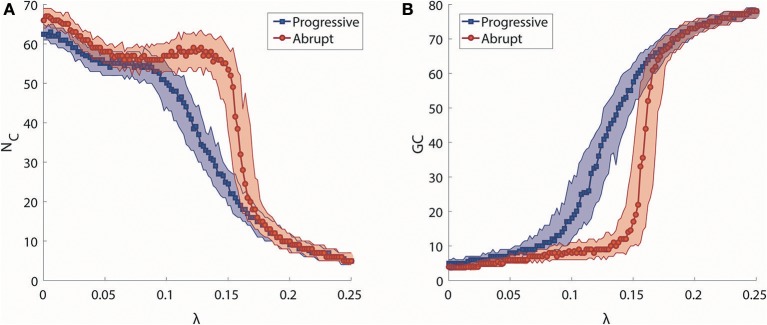
Analysis of synchronization clusters. **(A)** Change in the median number of synchronization clusters (N_C_) as a function of λ in progressive (blue) and abrupt (red) transitions. Colored area indicates the 25–75% values. **(B)** Change in the median size of a giant synchronization cluster (GC) as a function of λ in progressive (blue) and abrupt (red) transition. Colored area indicates the 25–75% values.

We investigated synchronization processes in the brain network at the level of clusters for progressive and abrupt transitions. The number of synchronization clusters, N_C_, and the size of giant synchronization cluster, GC, were examined to study how the clusters merge and how the size of the largest cluster develops during progressive and abrupt transitions (Figures 3A,B). As shown in Figure 3A, the evolution of N_C_ as a function of λ for two transitions is similar when λ is small (<0.086), but they show different cluster merging behaviors at higher values. In the progressive transition (blue), the N_C_ decreases slowly, whereas the N_C_ of the abrupt transition (red) is relatively preserved until λ ≅ 0.15 and thereafter sharply drops. The evolution of size of giant synchronization cluster, GC, for the two transitions is illustrated in Figure 3B. The size of GC in the progressive transition (blue) grows gradually, while the size of giant synchronization clusters of the abrupt transition (red) grows faster with constraints that make the largest synchronization cluster size bigger than 15 before λ = 0.148. Thus, the progressive transition follows the general synchronization path, in which a cluster is first centered around hub nodes attracting circumjacent peripheral nodes, and gradually grows into a dominant giant cluster (Gómez-Gardeñes et al., 2007). By contrast, for the abrupt transition, several smaller sized clusters are formed, but do not merge together until a certain coupling strength. At a critical point, they abruptly coalesce into big clusters (Zhang et al., 2014). In comparison to the progressive transition, the abrupt transition consistently demonstrates the delay of the major change and the sharp drop of the N_C_ with abrupt growth of the size of abrupt growth of the size of the GC.

### General relationship between structure and dynamics

We examined the global relationship between network structure (node degree) and node dynamics (local order parameter) as well as how it changes along with increasing coupling strength for progressive and abrupt transitions. The Spearman correlation between node degree and median r_i_ over 100 configurations was calculated to elucidate the global relationship.

**Figure 4:**
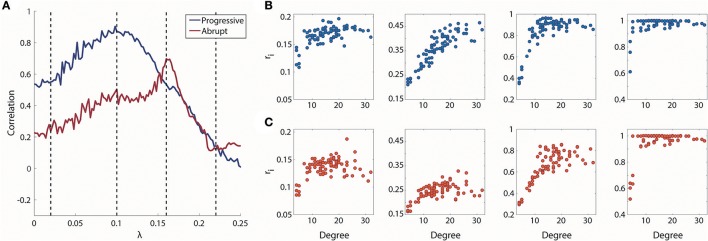
Relationship between degree and local order parameter r_i_ for coupling strength λ. **(A)** Spearman correlation between degree and median local order parameter r_i_ for coupling strength λ. Blue line indicates progressive transition and red line indicates abrupt transition. Black dotted vertical lines indicate λ = 0.02, 0.1, 0.16, and 0.22. Four representations of degree vs. r_i_ for λ = 0.02, 0.1, 0.16, and 0.22 (from left to right) in **(B)** progressive and **(C)** abrupt transition (Spearman correlation = 0.55, 0.87, 0.53, and 0.14 for gradual; Spearman correlation = 0.24, 0.50, 0.70, and 0.12 for abrupt transition).

The overall correlation values between node degrees and local order parameters of the progressive transition are higher than the abrupt transition in Figure 4A. During the progressive transition, the correlation increases until it has a maximum value at λ = 0.1. After that point, the correlation decreases until it has almost zero value. The relatively higher correlations of the progressive transition imply that the synchronization strengths of the brain regions during the progressive transition are more predictable and reflected at the individual node level. The correlation of the abrupt transition reaches a maximum (=0.70), which is delayed compared to the progressive transition and with a lower value than the progressive transition (=0.87).

### Dynamics of hub and peripheral nodes

**Figure 5:**
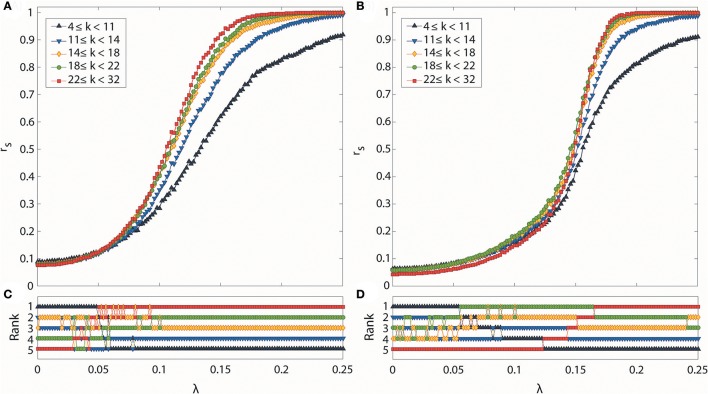
Synchronization level and synchronization rank for subgroups classified according to degree. Global synchronization monitored by median *D*_*ij*_ for all nodes i of each subgroup in **(A)** progressive and **(B)** abrupt transitions. Each colored line indicates a synchronization level of each subgroup classified with node degree k (black: 4 ≤ k < 11, blue: 11 ≤ k < 14, yellow: 14 ≤ k < 18, green: 18 ≤ k < 22, and red: 22 ≤ k < 32). Synchronization rank among subgroups in **(C)** progressive and **(D)** abrupt transitions. Each color is the same as denoted in **(A,B)**. In the progressive transition, subgroups with a higher degree are synchronized earlier. In the abrupt transition, the synchronization of hub nodes is suppressed below a certain value λ, but sharply increases after the critical point is reached.

We analyzed how hub and peripheral structures in the brain network are reorganized during the progressive and abrupt transitions as coupling strength increases (Figures 5A,B). We ranked the degree-classified subgroups in terms of the median r_s_ over 100 configurations for each subgroup (Figures 5C,D). The initial ranks were randomly given by the initial network configurations for both transitions. However, the ranks of subgroups are reorganized in distinctive ways as the coupling strength increases, depending on the type of transition. The reorganization among the subgroups takes place in a low and short coupling strength range (λ: 0.03–0.07) for the progressive transition, whereas it occurs in a relatively high and broad coupling strength range (λ: 0.064–0.168) for the abrupt transition.

During the progressive transition, the subgroups of higher degree go up to higher ranks of synchronization, while the subgroups of lower degree descend to the lower ranks of synchronization. At the end of the short and random reorganization process, the five subgroups have been arranged in descending order of degree with the descending order of synchronization. This reorganization process takes place before the major increase of the global order parameter of the brain network (Figure 2A), and before reaching the maximum correlation between node degrees and local order parameters (λ = 0.1) in Figure 3A.

The abrupt transition demonstrates a significantly different reorganization process compared to the progressive transition. The suppression of synchronization due to the initial network configuration makes the full ordered ranks delayed until λ = 0.168. The reorganization follows a systematic process, going up or down only one rank, rather than by discontinuous jumps of multiple steps. Interestingly, before the highest degree group occupies the top rank, the other subgroups already have established and maintained the ordered ranks (λ < 0.122). During this period, the highest degree group remains in the bottom rank, then rises to the higher rank in a step-wise fashion. The coupling strength at which the five subgroups were fully reorganized in ordered rank is similar to the coupling strength of the maximum correlation between node degrees and local order parameters in Figure 4A.

### Reconstitution of brain regions for progressive and abrupt transitions

**Figure 6:**
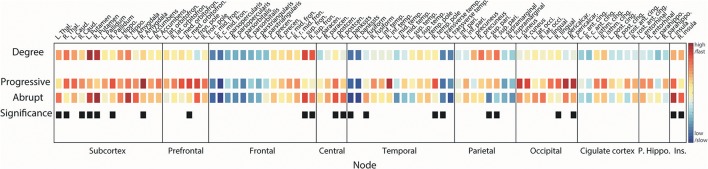
Reconstitution orders of regional brain structures. Squares in 1st line show node degrees of the region. Squares in 2nd (3rd) line are the reconstitution orders for progressive (abrupt) transition. Warmer (cooler) color indicates a higher (lower) degree or faster (slower) integration. Difference between first coupling strength values reaching local order parameter r_i_ > 0.2 and r_i_ > 0.8 was defined as the integration duration for each region. Median rank of integration durations over 100 configurations was used for ordering. Black squares in 4th line mark the regions that have statistically different reconstitution orders between progressive and abrupt transitions (Wilcoxon rank sum test; *p* < 0.05 considered significant). Name of brain regions was abbreviated for convenience (See Table S1 for full name).

In order to investigate the reconstitution processes in actual brain structures, we compared the ranks of τ_i_ of brain regions within each transition and for both transitions (Figure 6).

In accordance with the synchronization process we identified above (hub nodes are synchronized earlier than peripheral nodes), the higher degree brain regions recover faster compared with the lower degree regions in both transitions (Spearman correlation = −0.51, *p* < 0.001 for progressive transition; Spearman correlation = −0.59 and *p* < 0.001 for abrupt transition). This pattern is maintained even if the reconstitution duration of the abrupt transition is relatively shorter than the progressive transition. Most subcortical regions—including thalamus, caudate, putamen, pallidum, hippocampus, and accumbens—recover at early stages (reconstitution order <20, Table S1) in both transitions. Ranks of all regions for 100 configurations are presented in Figure S2. For progressive transitions, nodes in subcortical, prefrontal, and occipital regions reconstitute earlier than other regions (*p* < 0.05, significantly earlier than at least 5 regions). Recovery of central and insular regions is faster than the other regions in the abrupt transition (*p* < 0.05, significantly earlier than at least 5 regions). Hub nodes in frontal (bilateral sup. fron.) and parietal (bilateral sup. pari.) regions in progressive transition are relatively integrated at the same stages (rank = 15 and 17 for bilateral sup. fron., and rank = 12 and 11 for bilateral sup. pari.) but there is a significant difference of reconstitution order between them in the abrupt transition (rank = 4 and 8 for bilateral sup. fron., and rank = 30 and 24 for bilateral sup. pari., *p* < 0.05). Average ranks of sub-regions are provided in Figure S3.

Nodes with significantly different reconstitution order between progressive and abrupt transition are marked as black squares in the last line in Figure 6 (Wilcoxon rank sum test; *p* < 0.05). Recovery of bilateral putamen and left thalamus is faster in the abrupt transition. Reconstitution of the prefrontal area in the progressive transition seems to occur faster than the abrupt transition with a significant difference in right medial orbitofrontal cortex. Bilateral superior frontal regions are integrated faster in the abrupt transition whereas bilateral superior parietal regions are integrated faster in the progressive transition. Bilateral insular and bilateral post-central (primary somatosensory cortex) regions are relatively reconstituted at early stage in abrupt transition.

## Discussion

Synchronization of neural activities is an important condition for efficient information transmission among neural populations (Varela et al., 2001; Tononi, 2004; Melloni et al., 2007; Uhlhaas et al., 2009; Wang, 2010; Hipp et al., 2011; Plankar et al., 2013; Bressler and Richter, 2015). Anesthesia induces unconsciousness, fragmenting functional brain networks, and disrupting efficient information integration (Alkire et al., 2008; Lee et al., 2009; Boveroux et al., 2010; Ku et al., 2011; Schrouff et al., 2011; Schröter et al., 2012; Casali et al., 2013; Jordan et al., 2013; Lee U. et al., 2013; MacDonald et al., 2015), which is usually accompanied by global spatiotemporal desynchronization of the brain (Imas et al., 2006; Lee H. et al., 2013; Blain-Moraes et al., 2014; Liang et al., 2015; Palanca et al., 2015; Huang et al., 2016; Kim et al., 2016). However, after discontinuation of general anesthetics, the brain restores its activity spontaneously and with diverse patterns (Lee et al., 2011; Chander et al., 2014; Hight et al., 2014). Previous empirical data analysis has demonstrated distinctive evolution patterns of EEG for progressive/earlier or abrupt/delayed emergence from anesthesia. The characteristic evolution patterns of empirical EEG can potentially be explained by the patterns of progressive and abrupt synchronization transitions that were identified in this study of a neuroanatomically-informed model of the human brain. Notably, the scope of the current study is to simulate the macroscopic network of the whole-brain level. It should be differentiated from the previous state transition studies in mesoscopic networks at the neural population level (Steyn-Ross et al., 1999, 2001, 2004).

Progressive synchronization has been studied extensively with a focus on the effects of hub, modular structure, and global network topology in model and brain networks (Honey and Sporns, 2008; Kitzbichler et al., 2009; Breakspear et al., 2010; Gómez-Gardeñes et al., 2010; Cabral et al., 2011; Villegas et al., 2014; Hellyer et al., 2015; Schmidt et al., 2015; Váša et al., 2015; Finger et al., 2016). The hubs in scale-free networks dominate the synchronization process, whereas in random networks (which lack hub nodes), many individual nodes are synchronized earlier (Gómez-Gardeñes et al., 2007). In human brain networks, the hub-to-hub connections are critical for inter-modular synchronization and the perturbation of the rich club hubs significantly suppresses synchronization among the functional modules (Schmidt et al., 2015). Moreover, the location of each node in a network determines the temporal order of the synchronization process. For instance, the connector hub, which mediates several modular structures in a network, is synchronized at the last moment (Arenas et al., 2006). This is true across species; as one example, the hub areas in the cat brain consistently played a critical role in the synchronization process (Gómez-Gardeñes et al., 2010).

In contrast to the many studies of progressive synchronization, abrupt synchronization in a network has been investigated only recently with a series of studies focused on explosive synchronization (Gómez-Gardeñes et al., 2011; Leyva et al., 2013a,b; Li et al., 2013; Zhang et al., 2014, 2015). Since the key mechanism of explosive synchronization is to suppress the synchronization of hub nodes, we were able to predict the hub dominance in progressive synchronization. If hub structure is disrupted, there can be a significant change in the synchronization process.

In this study, we simulated emergence patterns in brain networks using the Kuramoto phase oscillator model applied to a human brain network. Altering the network configurations allowed us to model transitions at the individual node, cluster, and global network levels. We identified distinct patterns of progressive and abrupt synchronization transitions (progressive/early, abrupt/delayed). These synchronization phenotypes are consistent with behavioral phenotypes and related EEG patterns identified during progressive and abrupt emergence from unconsciousness (Lee et al., 2011; Chander et al., 2014; Hight et al., 2014). In addition, the simulation study sheds light on how regional brain functions reconstitute during progressive and abrupt emergence from anesthesia.

During progressive synchronization, the brain network is synchronized gradually from hub nodes attracting peripheral nodes and the synchronization cascade is triggered earlier with a lower coupling strength. The reorganization process at the subgroup level is completed and remains stable before major global change, which indicates that the brain network is sub-structurally already well-organized at the early stage of synchronization.

By contrast, during explosive patterns of synchronization, a network is synchronized discontinuously at a critical point with the delay of global integration (Zhang et al., 2014). For the abrupt transition, we used the V-shape relationship between node and frequency (i.e., hub nodes have higher and lower frequencies than central frequency at the same time) with large frequency disassortativity (higher frequency nodes tend to link with lower frequency nodes, or vice versa) to inhibit the synchronization of hub nodes (Leyva et al., 2013b; Li et al., 2013). Consequently, the suppression of hub synchronization prohibits the formation of giant clusters, allowing many small but disconnected clusters to grow until the network reaches the critical threshold where a small perturbation triggers the abrupt transition to global synchronization. Therefore, the global synchronization is delayed, but all clusters are combined at once in a single explosive unification of the brain network. In the reorganization process at the subgroup level, the delayed and slow reorganization of hub groups induces delayed synchronization throughout the brain. The reorganization of hub groups occurs with the change of individual node and global network levels, which means the brain network is not prepared to be organized at the sub-structural level in comparison with the gradual transition. The network configuration for the abrupt transition might mirror the different dose-dependent effects of anesthetic drugs on brain regions (Detsch et al., 1999; Liu et al., 2013; Sellers et al., 2013; Hutchison et al., 2014; Lv et al., 2016), a hypothesis that requires empirical confirmation.

Another novel finding in this study was that, in the correlation between node degree and local order parameter, progressive transitions have a larger correlation than abrupt transitions. This implies that the local dynamics of progressive synchronization processes are more predictable based on the brain network structure within a broad range of coupling strengths. The maximum peaks of correlation in both transitions indicate that the local order parameters are linearly arranged along with the node degrees before the formation of a giant synchronization cluster (Figures 3A, 4B,C). Notably, the maximum correlations between network structure and local dynamics during both transitions might reveal critical states in which the networks balance functional integration and segregation for the given conditions. Although the network reconfiguration processes are distinctive between progressive and abrupt transition, the principle of a higher degree node leading to higher local synchronization seems to be a necessary condition for triggering the global synchronization process in both transitions.

The simulations performed, based only on network principles of the two synchronization processes, yielded results that are consistent with the reconstitution of human brain functions from anesthesia. In both transitions, there was an early recovery of subcortical areas and a relatively late recovery of cortical areas, especially frontal and parietal areas. This is consistent with empirical findings using positron emission tomography data during recovery from propofol and dexmedetomidine sedation (Långsjö et al., 2012). In terms of differences between the two transitions, the earlier and bilateral recovery of the insula in the abrupt synchronization warrants further investigation, especially given roles in homeostasis, pain, and the coherent self.

## Limitations

This model study has many limitations. First, a simple coupled oscillatory model like Kuramoto limits interpretation because it can only capture the coarse-grained, large-scale synchronization process. Second, there are regional and temporal patterns of EEG in various frequency ranges during anesthesia and we are unable to explain complex EEG patterns with this model. We only took into account the alpha frequency band of EEG around 10 Hz to simulate the regional brain dynamics, but there are numerous other oscillations of relevance to consciousness and unconsciousness. More detailed models exhibiting a broad range of frequency spectrum will be important for further study. Third, the human brain network is neither a random, nor a scale-free network. It has a complex structure through which information transmission can be efficiently achieved with many types of hubs (Van Den Heuvel and Sporns, 2013). With this complicated structure, it is difficult to find the exact conditions for explosive synchronization. Therefore, the V-shape relationship between node degrees and frequencies as well as the large frequency disassortativity that we used are not unique methods to suppress the synchronization of hub nodes. Another possible network configuration suppressing the synchronization of hub nodes could alter the synchronization process. Fourth, despite our primary focus on modeling progressive and abrupt emergence, a mixed pattern of progressive and abrupt transitions empirically exists. Future study would be required to generalize our models toward a combined version of progressive and abrupt transitions. Fifth, we determined the hub nodes based only on the anatomical brain network structure. However, the effects of nodes on synchronization, even with the same anatomical node degree, could be different depending on their local network structure. Sixth, we used an anatomical human brain network parceled out into 82 nodes including cortical and subcortical regions. The finite-size effect of the network could have affected our results. To mitigate the finite size effect, we repeated the simulation 100 times and considered the averaged feature. Finally, we have established only loose associations between network principles of synchronization and behavioral or EEG phenotypes of recovery observed in humans recovering from anesthesia. Further work that studies synchronization processes in humans during the reconstitution of consciousness and cognition will be important to validate these findings.

## Conclusions

This model study demonstrated that progressive and abrupt synchronization transitions in a human brain network can occur based on network principles alone. Distinctive characteristics of network synchronization processes appear to match progressive and abrupt emergence patterns from unconsciousness based on behavior and EEG patterns. The characteristic network reconstitution processes observed at the individual node, cluster, and global network levels suggest underlying mechanisms for how regional brain functions are reconstituted during the progressive and abrupt emergence from the unconscious state, providing a theoretical foundation for further studies.

## Author contributions

MK performed research and wrote the paper. SK and GM interpreted data and contributed to writing the paper. UL conceived of the study, interpreted the data, and wrote the paper.

### Conflict of interest statement

The authors declare that the research was conducted in the absence of any commercial or financial relationships that could be construed as a potential conflict of interest.
