# Supplementary material for: Relationship of Topology, Multiscale Phase Synchronization, and State Transitions in Human Brain Networks
Source: Front Comput Neurosci. 2017 Jun 30;11:55. doi: 10.3389/fncom.2017.00055 (PMC5492767; doi:10.3389/fncom.2017.00055)
Supplement: Supplementary file 1 [file Presentation1.PDF]

Supplementary

Figure S1. Robustness test for various network configurations

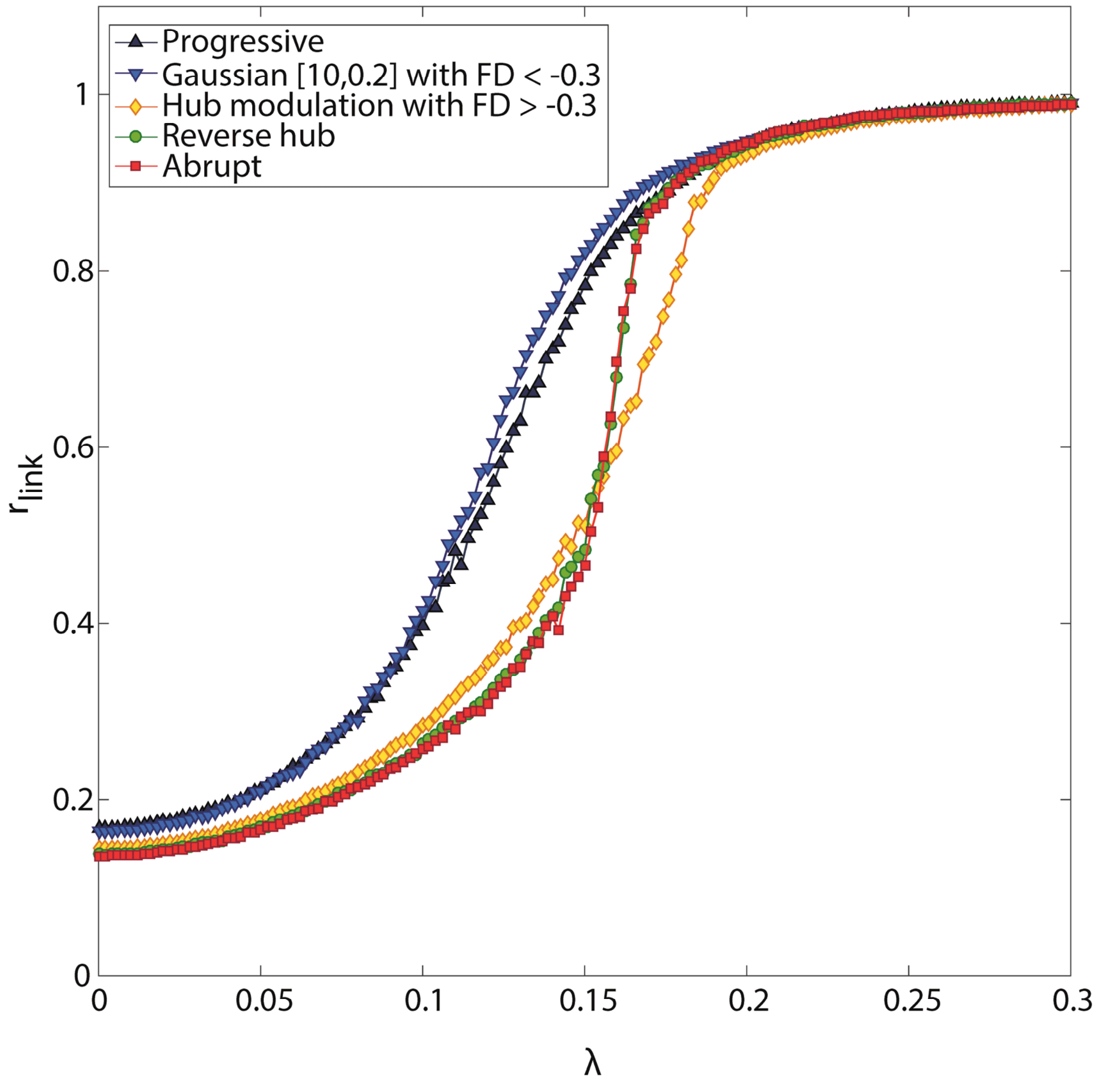

Figure S1. Global synchronization for progressive transition (black), abrupt transition (red) and transitions with different frequency configurations. Median global order parameters for 100 frequency configurations were calculated for each transition. Gaussian distribution with mean 10.2 Hz and variance 0.2 Hz with frequency disassortativity is smaller than -0.3 (blue) was tested for the robustness of distinctive frequencies of hub nodes. The same distinctive hub node selection with less disassortative frequency (yellow) was tested for the robustness of frequency disassortativity values. Finally, reversed distinctive frequencies with hub nodes (green) were tested.

Figure S2. Region ranks of 100 configurations for progressive and abrupt transitions

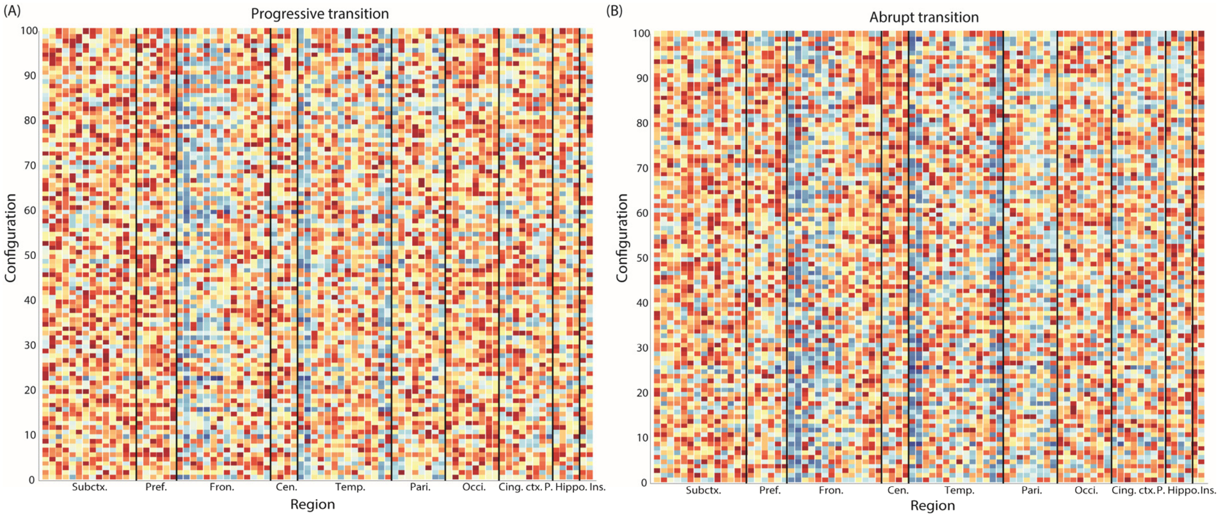

Figure S2. Region ranks of 100 configurations for (A) progressive and (B) abrupt transitions. Warmer (cooler) color indicates the faster (slower) integration. Regions are classified with subcortex (Subctx.), prefrontal (Pref.), frontal (Fron.), central (Cen.), temporal (Temp.), parietal (Pari.), occipital (Occi.), cingulate cortex (Cing. Ctx.), parahippocampal cortex (P. Hippo.), and insula (Ins.)

Figure S3. Average integration order (rank) of brain regions

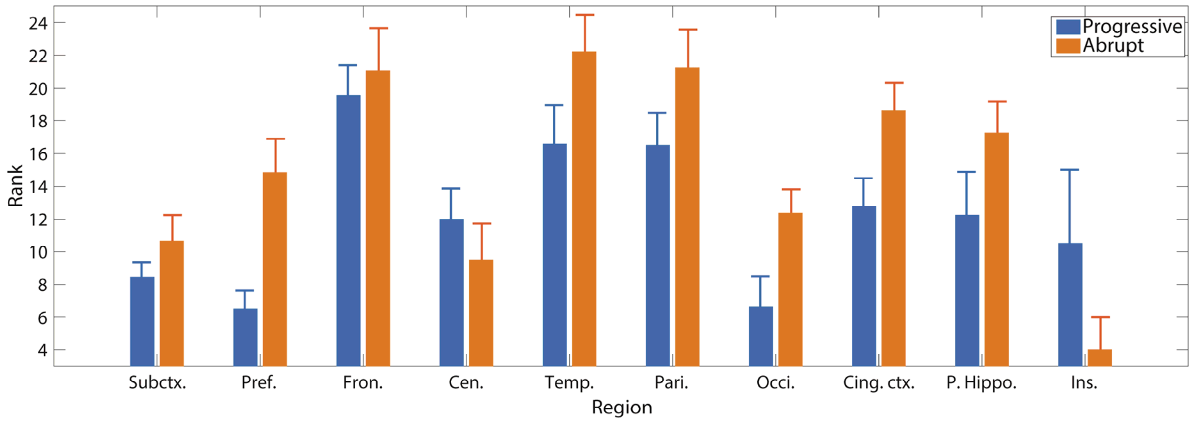

Figure S3. Average integration order of brain regions. Average of median rank over the brain regions within subcortex (Subctx.), prefrontal (Pref.), frontal (Fron.), central (Cen.), temporal (Temp.), parietal (Pari.), occipital (Occi.), cingulate cortex (Cing. Ctx.), parahippocampal cortex (P. Hippo.), and insula (Ins.) is illustrated (Mean ± Standard error).

Table S1. Brain region name and median rank for two transitions

	Region name	Abbreviation	Rank (progressive)	Rank (abrupt)
Subcortex	L. Thalamus	L. Thal.	14	8
	R. Thalamus	R. Thal.	6	17
	L. Caudate	L. Caud.	6	12
	R. Caudate	R. Caud.	9	19
	L. Putamen	L. Putamen	11	3
	R. Putamen	R. Putamen	13	1
	L. Pallidum	L. Pallidum	10	19
	R. Pallidum	R. Pallidum	5	14
	L. Hippocampus	L. Hippo.	10	9
	R. Hippocampus	R. Hippo.	7	2
	L. Amygdala	L. Amygdala	9	6
	R. Amygdala	R. Amygdala	1	13
	L. Accumbens	L. Accumbens	10	13
	R. Accumbens	R. Accumbens	7	13
Prefrontal	L. lateral orbitofrontal	L. lat. orbitofron.	11	20
	R. lateral orbitofrontal	R. lat. orbitofron.	8	16
	L. medial orbitofrontal	L. med. orbitofron.	5	19
	R. medial orbitofrontal	R. med. orbitofron.	4	15
	L. frontal pole	L. frontal pole	4	6
	R. frontal pole	R. frontal pole	7	13
Frontal	L. caudal middle frontal	L. c. mid. fron.	27	32
	R. caudal middle frontal	R. c. mid. fron.	31	34
	L. parsopercularis	L. parsopercularis	21	28
	R. parsopercularis	R. parsopercularis	24	29
	L. parsorbitalis	L. parsorbitalis	27	31
	R. parsorbitalis	R. parsorbitalis	22	27
	L. parstriangularis	L. parstriangularis	24	26
	R. parstriangularis	R. parstriangularis	21	19
	L. precentral	L. precen.	14	13
	R. precentral	R. precen.	12	17
	L. rostral middle frontal	L. r. mid. fron.	10	15
	R. rostral middle frontal	R. r. mid. fron.	9	12
	L. superior frontal	L. sup. fron.	15	4
	R. superior frontal	R. sup. fron.	17	8
Central	L. paracentral	L. paracen.	16	15

	R. paracentral	R. paracen.	12	11
	L. postcentral	L. postcen.	7	5
	R. postcentral	R. postcen.	13	7
**Temporal**	L. bankssts	L. bankssts	29	34
	R. bankssts	R. bankssts	30	34
	L. fusiform	L. fusiform	17	11
	R. fusiform	R. fusiform	9	15
	L. inferior temporal	L. inf. temp.	11	16
	R. inferior temporal	R. inf. temp.	2	15
	L. middle temporal	L. mid. temp.	20	28
	R. middle temporal	R. mid. temp.	13	25
	L. superior temporal	L. sup. temp.	16	24
	R. superior temporal	R. sup. temp.	13	14
	L. temporal pole	L. temporal pole	13	13
	R. temporal pole	R. temporal pole	5	19
	L. transverse temporal	L. transverse temp.	28	30
	R. transverse temporal	R. transverse temp.	26	33
**Parietal**	L. inferior parietal	L. inf. pari.	18	19
	R. inferior parietal	R. inf. pari.	11	13
	L. precuneus	L. precuneus	19	15
	R. precuneus	R. precuneus	13	15
	L. superior parietal	L. sup. pari.	12	30
	R. superior parietal	R. sup. pari.	11	24
	L. supramarginal	L. supramarginal	25	26
	R. supramarginal	R. supramarginal	23	28
**Occipital**	L. cuneus	L. cuneus	3	10
	R. cuneus	R. cuneus	4	13
	L. lateral occipital	L. lat. occi.	16	10
	R. lateral occipital	R. lat. occi.	13	8
	L. lingual	L. lingual	7	18
	R. lingual	R. lingual	6	19
	L. pericalcarine	L. pericalcarine	2	9
	R. pericalcarine	R. pericalcarine	2	12
**Cigulate cortex**	L. caudal anterior cingulate	L. c. ant. cing.	18	23
	R. caudal anterior cingulate	R. c. ant. cing.	20	24
	L. isthmus cingulate	L. isthm. cing.	8	14
	R. isthmus cingulate	R. isthm. cing.	6	13
	L. posterior cingulate	L. post. cing.	13	22

	R. posterior cingulate	R. post. cing.	16	17
	L. rostral anterior cingulate	L. r. ant. cing.	10	23
	R. rostral anterior cingulate	R. r. ant. cing.	11	13
**Parahippocampal cortex**	L. entorhinal	L. entorhinal	9	12
	R. entorhinal	R. entorhinal	9	19
	L. parahippocampal	L. parahippo.	20	17
	R. parahippocampal	R. parahippo.	11	21
**Insula**	L. insula	L. insula	6	2
	R. insula	R. insula	15	6
